# Flexible Antagonist versus Agonist Flare Protocol in Women above 40 Undergoing IVF, A retrospective Cohort Study

**DOI:** 10.5935/1518-0557.20220064

**Published:** 2023

**Authors:** Elsayed Ali Farag Hassabelnabi, Mohammad Ahmad Badreldin, Mohammad Atef Behery, Kamaleldin Abdullah Rageh, Eman Ahmed Ali

**Affiliations:** 1 Almana General Hospital, IVF unit, Eastern Provence, Saudi Arabia; 2 Department of Obstetrics and Gynecology, Faculty of medicine, Al-Azhar University, Cairo, Egypt; 3 Assisted Reproduction Unit, The International Islamic Center for Population Studies and Research, Al-Azhar University, Cairo, Egypt

**Keywords:** GnRH antagonist protocol, flare protocol, IVF outcome, controlled ovarian stimulation

## Abstract

**Objective:**

Several strategies have been proposed for ovarian stimulation in older women, such as using an increased daily dose of gonadotropins (300-450 IU per day) with GnRH agonist (long or micro dose flare protocols), or using GnRH antagonist protocols. The objective of this study is to compare the efficacy of flexible GnRH antagonist protocol and GnRH agonist flare - pituitary block protocols for ovarian stimulation in women above 40 years old undergoing IVF.

**Methods:**

This study was performed between January 2016 and February 2019. One hundred and fourteen women aged between 40 and 42 years who underwent IVF were divided into two groups; group I were treated by Flexible GnRH antagonist protocol (Antagonist group, n=68); and group II were treated by Flare GnRH agonist protocol (Flare group, n=46).

**Results:**

Patients treated with the antagonist protocol had a significantly lower cancellation rate when compared with patients treated with flare agonist protocol (10.3% vs. 21.7%, p value 0.049). The other parameters evaluated did not show statistically significant differences.

**Conclusions:**

Our finding showed that both Flexible antagonist and Flare agonist protocols had comparable outcomes, with lower cycle cancellation rates for older patients treated with the antagonist protocol.

## INTRODUCTION

The reproductive capacity of women in their late reproductive years is a subject of great interest in the field of reproductive medicine (Speroff & Fritz, 2012). Women over 40 years of age undergoing IVF/ICSI procedures represent the most rapidly growing population in the world (Gleicher *et al*., 2016). It was estimated that 19% of all women using ART in the United States, 15% of women undergoing IVF in Europe and 10% of IVF cycles in the middle east were for women aged over 40 years (CDC, 2021; Nyboe Andersen *et al*., 2009; Mansour & Abou Setta, 2006).

Fecundity and reproductive performance progressively declined with advanced maternal age; this is mainly attributed to diminished ovarian reserve and decreased egg quality. Age is associated with poor IVF outcome, as evidenced from the IVF registry data, and the expectancy of pregnancy per cycle started is markedly lower in older than in younger women. Furthermore, women of advanced age attending IVF procedures have higher degrees of ovarian resistance to trophic hormone stimulation, and also a significantly higher rate of poor responders and cycle cancellations than in the general population (Hourvitz *et al*., 2009; SART & ASRM, 2004; Dal Prato *et al*., 2005; Pellicer *et al*., 1994). Ovarian stimulation in those potentially poor responder women is a very important step in the success of IVF procedure. Several strategies have been proposed for ovarian stimulation, such as using an increased daily dose of gonadotropins (300-450 IU per day) with GnRH agonist (long or micro dose flare protocols), or using GnRH antagonist protocols (Mahutte & Arici, 2007).

The aim of this study was to compare the effectiveness of the flexible antagonist and the flare agonist protocols in ovarian stimulation for potentially poor responders, advanced age women undergoing IVF treatment.

## MATERIAL AND METHODS

The current study was a retrospective cohort, in which the medical records of all IVF patients over 40 years treated at the IVF unit at Almana General Hospital, Eastern Provence, KSA between January 2016 and February 2019 were analyzed *after an approval from the local ethical board*. The women aged between 40-42 years and who had basal FSH levels lower than 10 mIU/ml, underwent fresh embryo transfer; and those who had no history of poor response were eligible for the study.

We used two pituitary block protocols for controlled ovarian stimulation. In the GnRH antagonist protocol (antagonist group n=68), the patients were treated with 375 IU of recombinant FSH (Gonal-F, Serono, Modugno (BA), Italy) started on day-2 of the cycle, and GnRH antagonist (Cetrotide, Serono, Switzerland) (0.25 mg/day s.c injection) was introduced when the growing follicles became 14 mm in diameter and continued till the day of HCG triggering. In the flare agonist protocol (agonist group n=46), the patients were treated with GnRH-agonist (Decapeptyl, Ferring pharmaceuticals, Kiel, Germany) (0.1 mg/ day s.c injection), starting from the day-1 of the cycle, followed by 375 IU of recombinant FSH (Gonal-F, Serono) from the day-2. In both groups, the recombinant-FSH dose was adjusted based on the individual ovarian response. Recombinant HCG (Ovitrelle, Serono, Italy) (250 MCG) was adminstered i.m. when there were at least two follicles of 18 mm in diameter. Ultrasound-guided oocyte retrieval was performed 36 hours later. Conventional insemination and in-vitro fertilization were performed as indicated. Two to three fresh embryos (grades 1 or 2) were transferred on day 3. Progesterone supplementation (Cyclogest 400 mg transvaginal BID) was given to all women from day-1 after oocyte retrieval and coninued until the pregnancy test. Positive pregnancy was defined as a serum HCG concentration of 5 IU/l on day-16 after transfer. Clinical pregnancy was defined as the presence of a gestational sac and visualization of fetal heartbeat by ultrasound 2 weeks later. Data were collected and analyzed using the Statistics Package for Social Sciences (SPSS) version 22 for windows program (SPSS, Inc., Chicago, IL, USA). Chi-squared or the Fisher’s exact tests for categorical variables and the unpaired two-way Student’s t-test for continuous factors were used as appropriate. A *p*-value of <0.05 was considered significant.

### Ethical Approval

The study was approved by the local ethical board at Almana General Hospital.

## RESULTS

One hundred and fourteen cases were eligible for the study, 68 patients (59%) were treated by the GnRH antagonist protocol and 46 patients (41%) were treated by the GnRH agonist flare protocol. Patients in both groups showed no significant difference regarding the basic characteristics; (age, BMI), basal hormonal profile (FSH, LH), antral follicle count (AFC), duration of infertility, type and causes of infertility (Table 1).

**Table 1:** Antagonist x Flare up: Clinical data for both group.

Variables	Antagonist group (n=68)	Flare group (n=46)	*p* value
**Age (years)**	41.1±0.78	40.8±0.76	0.735
**BMI (Kg/m^2^)**	28.5±3.1	28.7±2.9	0.906
**FSH (mIU/ml**	5.7±1.08	6.3±2.02	0.104
**LH (mIU/ml)**	4.2±1.8	4.7±2.3	0.173
**AFC**	4.2±3.15	7.4±4.1	0.956
**Duration of infertility (years)**	8.5±4.9	7.4±4.1	0.223
**Cause of infertility** **Female factor** **Male factor** **Both male and female** **Unexplained**	21 (30.9%) 39 (57.4%) 6 (8.8%) 2 (2.9%)	11 (23.9%) 27 (58.7%) 7 (15.2%) 1 (2.2%)	0.676
**Type of infertility** **Primary** **Secondary**	37 (54.4%) 31 (54.6%)	24 (52.2%) 22 (47.8%)	0.482

Mean ±SD was used for representation of quantitative data while number and percentage was used for representation of categorical data.

Student t-test was used for comparison of quantitative data.

Chi-square test was used for comparison of categorical data.

**Figure 1:**
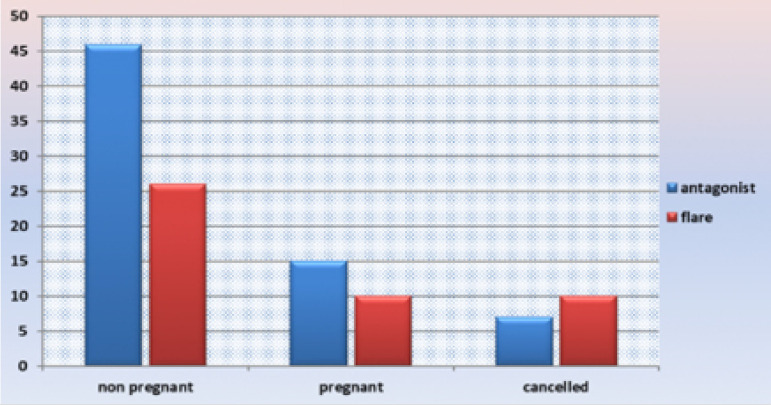
IVF trial outcome.

Results of the IVF trials were presented in Table 2; there were no significant differences between both groups vis-a-vis duration of stimulation, FSH consumption dose, number and quality of retrieved oocytes, number and quality of transferred embryos. Also, there was no statistical difference regarding the pregnancy rate, either per cycle started or per transfer between both groups (Figure 1, Table 2). The cycle cancellation rate was significantly lower in the antagonist group when compared to the flare group (10.3 *vs*. 21.7, *p*=0.049).

**Table 2:** Antagonist x Flare up: Outcome of IVF between both group.

Variables	Antagonist Group (n=68)	Flare group (n=46)	*p* value
**Duration of stimulation**	10.2±1.7	10.7±1.8	0.203
**Total dose of FSH**	3967.6±1230.7	4222.6±1103.4	0.261
**Number of retrieved oocytes**	5.2±4.5	5.9±4.4	0.454
**Number of M2 oocytes**	3.8±3.06	4.4±2.8	0.347
**Number of fertilized oocytes**	2.6±1.9	2.9±2.3	0.560
**Grade 1,2 embryos**	2.6±1.7	3.0±2.0	0.409
**Number of transferred embryos**	2.2±1.02	2.1±0.9	0.809
**Day of embryo transfer**	2.7±0.4	2.9±0.7	0.139
**Pregnancy rate per cycle**	15/68 (22%)	10/46 (21/7%)	0.233
**Pregnancy rate per transfer**	15/64 (23%)	10/41 (24.3%)	0.198
**Cancellation rate**	7/68 (10.3%)	10/46 (21.7%)	0.049^*^

* Significant if *p* ≤ 0.05

Mean ±SD was used for representation of quantitative data while number and percentage was used for representation of categorical data.

Student t-test was used for comparison of quantitative data.

Chi-square test was used for comparison of categorical data.

**Figure 2:**
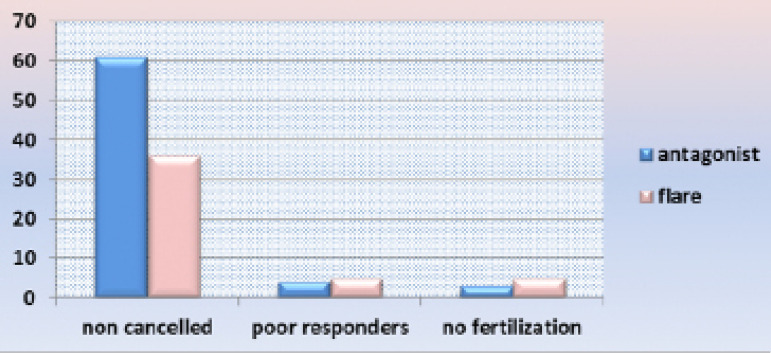
Causes of cycle cancellation.

Causes of cycle cancellation were presented at Figure 2: in the antagonist group, 4 cases were cancelled due to poor response and 3 cases due to no-fertilization while in the flare group, 5 cases were cancelled due to poor response and 5 cases due to no-fertilization.

## DISCUSSION

Infertile women of advanced age are unique group of patients with reduced fertility, mainly due to reduction in oocyte number and quality (Tsafrir *et al*., 2009). Treatment of this group of patients who are undergoing IVF treatment remains a challenge. Various treatment modalities have been adopted to increase ovarian response to fertility treatment in those patients; at the moment there is no single and effective established protocol. Given the low oocyte yield, it is essential to determine the best possible stimulation protocol for these patients.

In this retrospective study, we tried to investigate the feasibility of using the 2 most common pituitary block protocols for ovarian stimulation in this group of women with advanced age and sub-optimal fertility potential, the flexible GnRH antagonist and Flare up short agonist protocols.

The rationale behind GnRH antagonist regimen is to avoid the profound suppression of endogenous FSH and LH in the early follicular phase, at the stage of follicular recruitment; and thereby improving cycle outcome in poor responders, and the rationale behind the flare agonist protocol is minimizing the ovarian suppression of the long GnRH-a protocol, while getting the benefits of the flare effect on follicular recruitment.

Our study included 114 patients above 40 years of age, who received either the GnRH antagonist protocol (n=68) or the GnRH-a flare protocol (n=46). The patients in both groups had similar baseline characteristics, which makes the study more prone to compare outcomes between the two groups (Table 1).

Our data showed that there were no statistically significant differences between both groups regarding the duration of stimulation, FSH consumption dose, number and quality of retrieved oocytes or transferred embryos. Also, there was no statistical difference as to pregnancy rate, either per cycle started or per transfer between both groups. Prior randomized and nonrandomized studies have yielded varied results.

Several reports found that the antagonist and the flare protocols yielded similar results. Berin *et al*. (2010), in a retrospective chart review of 113 patients had achieved excellent and comparable pregnancy and live birth rates in poor responders of advanced reproductive age with the use of either GnRH antagonist or flare protocol.

Vollenhoven *et al*. (2008) did a large retrospective study for 1608 stimulation cycles to answer the question: Is there an ideal stimulation regimen for IVF for poor responders, and does it change with age? This large retrospective study of ‘poor’ responders has not shown a difference in pregnancy rates/initiated cycle between different stimulation regimens. Kahraman *et al*. (2009) did a prospective, randomized, clinical trial to compare the efficacy of microdose GnRH agonist (GnRH-a) flare-up and multiple dose GnRH antagonist protocols, in patients who have a poor response to a long luteal GnRH-a protocol; and concluded that microdose GnRH-a flare-up protocol and multiple dose GnRH antagonist protocol seem to have similar efficacy in improving treatment outcomes of poor responder patients. Madani *et al*. (2012) compared the efficacy of different stimulation protocols on pregnancy outcomes in poor responders undergoing IVF and suggested that the application of different protocols in poor responder patients seems to have similar efficacy in improving clinical outcomes such as implantation, pregnancy rates and cancellation rates. Hu *et al*. (2014) demonstrated that there is no difference in clinical outcomes such as clinical pregnancy rate and implantation rate between different protocols used for ovarian stimulation in poor responders.

On the other hand, many studies favored the use of the GnRH agonist flare protocol. Demirol & Gurgan (2009) concluded that the short agonist protocol seems to have a better outcome in poor responders than the multiple dose antagonist protocol, with a significantly higher number of mature oocytes retrieved and implantation rates. Similarly Malmusi *et al*. (2005) reported that the short agonist protocol appears to be more effective than the GnRH antagonist protocol in terms of mature oocytes retrieved fertilization rate and top-quality embryos transferred. Also Schimberni *et al*. (2016) showed that the short GnRH agonist protocol with its flare-up effect should be the first choice in poor responder women, especially in cases of women 40 years old or more, whereas the flexible GnRH antagonist protocol seems to be less effective in these patients.

However Sunkara *et al.* (2014) reported that in poor responders, the long GnRH-agonist and flexible GnRH-antagonist protocols worked better than the short GnRH agonist protocols in terms of oocytes harvested and total dose of gonadotropins administered. Also, Mahutte & Arici (2007) concluded that GnRH antagonist provides the advantages of shorter duration of stimulation with reduced gonadotrophin requirements. Lainas *et al*. (2008) reported that the flexible GnRH antagonist protocol is associated with significantly higher ongoing pregnancy rates when compared with the flare up GnRH agonist protocol in poor responders.

In our study, women treated by the antagonist protocol showed lower cycle cancellation rates (10.3 *vs*. 21.7, *p*=0.049) than those treated by the Flare agonist protocol. That finding was consistent with Ibrahim *et al*. (2011), who reported higher cancellation rates in the agonist group than in the antagonist group, but inconsistent with Mohamed *et al*. (2005), who revealed a significant higher cycle cancellation rate, and less patients having embryo transfer in the antagonist group.

## CONCLUSION

Our finding showed that both flexible antagonist and flare agonist protocols had comparable outcomes with lower cycle cancellation rates for older patients treated with the antagonist protocol. Controversies are raised in the literature and the authors recognize that additional studies are needed to confirm such superiority or the equivalence between protocols.

**Limitations of the study:** being a retrospective which could not be randomized.
